# The role of androgen deprivation therapy on biochemical failure and distant metastasis in intermediate-risk prostate cancer: effects of radiation dose escalation

**DOI:** 10.1186/s12885-015-1180-6

**Published:** 2015-03-27

**Authors:** Michelle S Ludwig, Deborah A Kuban, Xianglin L Du, David S Lopez, Jose-Miguel Yamal, Sara S Strom

**Affiliations:** Department of Radiology, Baylor College of Medicine, One Baylor Plaza, MS: BCM360 Room 165B, Houston, TX 77030 USA; Department of Radiation Oncology, University of Texas MD Anderson Cancer Center, Houston, TX 77030 USA; Department of Epidemiology, University of Texas MD Anderson Cancer Center, Houston, TX 77030 USA; Division of Epidemiology, Human Genetics, and Environmental Sciences, University of Texas School of Public Health, Houston, TX 77030 USA; Division of Biostatistics, University of Texas School of Public Health, Houston, TX 77030 USA

**Keywords:** Prostate cancer, Dose escalation, Androgen deprivation therapy, Intermediate risk prostate cancer

## Abstract

**Background:**

To determine whether the effect of androgen deprivation therapy (ADT) on the risk of biochemical failure varies at different doses of radiation in patients treated with definitive external beam radiation for intermediate risk prostate cancer (IRPC).

**Methods:**

This study included 1218 IRPC patients treated with definitive external beam radiation therapy to the prostate and seminal vesicles from June 1987 to January 2009 at our institution. Patient, treatment, and tumor information was collected, including age, race, Gleason score, radiation dose, PSA, T-stage, and months on ADT.

**Results:**

The median follow-up was 6 years. A total of 421(34.6%) patients received ADT, 211 (17.3%) patients experienced a biochemical failure, and 38 (3.1%) developed distant metastasis. On univariable analyses, higher PSA, earlier year of diagnosis, higher T-stage, lower doses of radiation, and the lack of ADT were associated with an increased risk of biochemical failure. No difference in biochemical failure was seen among different racial groups or with the use of greater than 6 months of ADT compared with less than 6 months. On multivariate analysis, the use of ADT was associated with a lower risk of biochemical failure than no ADT (HR, 0.599; 95% CI, 0.367-0.978; P < 0.04) and lower risk of distant metastasis (HR, 0.114; 95% CI, 0.014-0.905; P = 0.04).

**Conclusions:**

ADT reduced the risk of biochemical failure and distant metastasis in both low- and high dose radiation groups among men with intermediate-risk PCa. Increasing the duration of ADT beyond 6 months did not reduce the risk of biochemical failures. Better understanding the benefit of ADT in the era of dose escalation will require a randomized clinical trial.

## Background

The addition of Androgen Deprivation Therapy (ADT) to radiation therapy for locally advanced prostate cancer has demonstrated an improvement in local control and overall survival benefit in a number of randomized controlled trials [1-6]. Many of these trials were conducted in an era where lower doses of radiation were used and when patients were not evaluated in the risk groups that are now used to make clinical decisions. In all of the described trials that established the need for ADT with external beam radiation, a dose of 70 Gy or less was used. In 2002, results from a randomized trial by Pollack et al., showed that a 78 Gy dose improved survival and other several similar dose-escalation studies changed the recommended practice patterns by increasing the dose of prostate radiation [7-10]. In the face of this new standard of higher radiation doses, there is a need to evaluate the benefit of adding ADT in terms of optimal patient selection and optimal timing and duration of ADT.

ADT can cause adverse physical and psychological side effects in patients, such as decrease in muscle mass, increase in diabetes, decrease in bone density, depression, loss of libido, and others, so its use must be carefully balanced between benefit and risk [11,12]. Current National Comprehensive Cancer Network (NCCN) guidelines reflect this uncertainty by recommending “consideration of 4-6 months of ADT” if radiation therapy is given as definitive treatment for intermediate and high risk prostate cancer [13]. Given the adverse physical and quality of life effects of ADT and the unknown benefit of ADT in the era of radiation dose escalation, an evaluation of its benefit is needed. We conducted a retrospective clinical review of prostate cancer patients to determine whether the effect of ADT on the risk of biochemical failure and distant metastasis was the same at different doses of radiation in intermediate risk prostate cancer and whether the duration or timing of ADT resulted in improved outcomes.

## Methods

### Patient selection and pretreatment evaluation

This study included intermediate risk prostate cancer (defined according to NCCN criteria) patients who were treated at our institution with definitive external beam radiation therapy from June 1987 to January 2009 [13]. The proposal approval was granted by the University of Texas Health Science Center at Houston Committee for The Protection of Human Subjects #HSC-SPH-12-0475. The data were collected under the MD Anderson Cancer Center IRB as Protocol RCR02-127. All patients had biopsy-proven adenocarcinoma of the prostate with no metastatic disease at the time of diagnosis. The initial evaluation consisted of a history and physical, digital rectal exam to evaluate tumor stage (based on the 1992 American Joint Committee on Cancer staging system), serum PSA measurement, and biopsy with Gleason histologic grading. The bone scans and pelvic computerized tomography for staging were performed if the patient’s pretreatment PSA was ≥10 or Gleason score was ≥8.

### Treatment

All patients were treated with definitive external beam radiation therapy to the prostateand seminal vesicles. Prior to 2000, conventional four-field techniques were used with doses prescribed to the isocenter. After 2000, intensity-modulated radiation therapy was used to treat the prostate and seminal vesicles. Lymph nodes were not included in the clinical target volume. Radiation prescription doses ranged from 60 to 78 Gy, depending on the year of treatment. ADT was delivered either as total androgen blockade or a lutenizing hormone releasing hormone (LHRH) agonist alone, given at the discretion of the treating radiation oncologist.

### Follow-up and endpoints

Follow-up evaluation consisted of digital rectal examination and serum PSA measurements every 3-6 months for the first two years, then every six months for the next 3 years, and then annually after five years. Their medical records were analyzed in a retrospective fashion and institutional approval was received prior to initiating the study. The biochemical failures were coded by the “Phoenix” definition, or a rise in ≥2 ng/mL above the lowest PSA achieved after treatment, with the actual date of failure coded as the date of the PSA test [14]. Patients lost to follow-up were censored at the last visit. The interval to biochemical failure was calculated from the completion date of radiation therapy. Metastatic failures were outside of the pelvis, in nonregional lymph nodes, bone, or other places. Coding of metastatic disease was based on chart review.

### Statistical analysis

Descriptive measures were calculated for all variables, and the patients were divided into ADT and no ADT groups. Univariable analyses were conducted to determine the relationship between all variables and the outcome of biochemical failure or distant metastasis, using Cox proportional hazards models. Comparisons between the use of ADT and the other variables were assessed using a *t*-test. All variables were analyzed as continuous variables except for radiation dose, which was considered a binary variable (low dose radiation was defined as receiving < = 70 Gy, and high dose radiation was defined as > 70 Gy) and ADT (yes or no) and timing of ADT (neoadjuvant or adjuvant). Five and ten year rates of biochemical failure were calculated for the overall and four major groups. Radiation dose was evaluated for effect modification by both addition of an interaction term in the models and by stratification by dose of radiation. After stratification, the log-rank test for homogeneity of survival curves was used to test the difference in effect of ADT among the two strata. Multivariable Cox proportional hazards models were constructed to include possible confounders and effect modifiers when appropriate, and variables were removed from the model one by one to evaluate the change in hazards of the main effect variables. If a significant change was noted in the main outcome variable being tested (about 10-20%), the variable being tested remained in the model as a confounder. The proportional hazards assumption was tested for each variable in the model.

In the absence of definitive guidelines for placing patients on ADT, the decision is left to the treating physician. As such, this decision is likely to be influenced by patient factors (e.g., age and comorbidity) and tumor factors (PSA, t-stage, Gleason score), which are all known to contribute to outcomes of biochemical, local, and distant failures. In order to address this selection bias, propensity score analysis was utilized16. In our application, the propensity score estimates the conditional probability of a patient receiving a hormonal treatment given other covariates. To create the scores, a logistic model was used to estimate the probability of receiving ADT. Gleason Score, age, year of diagnosis, T stage, and pretreatment PSA were included into the multivariable logistic regression model, as these factors had been decided a priori to affect a clinician’s decision to place a patient on ADT, and variable selection was conducted using a backwards stepwise procedure. The propensity score-adjusted result has been shown to remove 90% of the bias in a continuous distribution [15]. Matching on the propensity score and using the propensity score as a covariate in the Cox model were used for the outcome of biochemical failures. Matching was then conducted; a 1:1 matched pair design without replacement was used to account for the nonrandom treatment allocation. Case patients (with ADT) were matched with control patients (no ADT). Since matching results in a violation of the independence assumption for a Cox model, a frailty term was used for the matched pairs in creation of the model.

## Results and discussion

A total of 1218 patients with intermediate risk prostate cancer were included in our study. Table 1 shows the characteristics of the patients by ADT status. The mean age for all patients was 68.5 years. Median follow up was 6 years. A total of 421 (34.6) patients received adjuvant ADT, with 211 (17.3%) patients experiencing a biochemical failure, and (3.1%) experiencing distant metastasis. Five year rates of biochemical failure were 9.7% and ten year rates were 16.1%. Of the patients who received ADT, a total of 271 (64.4%) patients received 6 months or less, and 150 (35.6%) patients were on ADT for longer than 6 months. Radiation dose was divided into low (≤70 gy, n = 418, 34.3%) or high (>70 gy n = 800, 65.7%) for all analyses. A majority (76%) of the patients were white. A total of 114 (14.3%) of patients who did not receive concurrent ADT subsequently were placed on salvage hormonal therapy for failures compared to 8 patients (1.9%) who initially received concurrent ADT.

**Table 1:** **Distribution of baseline variables by concurrent ADT status**

**Variable**	**ADT**	**No ADT**	**Total**	**p-value**	**no ADT**	**p-value**
	**(cases)**	**(unmatched controls)**		**(unmatched)**	**(matched controls)**	**(matched)**
	**n = 421**	**n = 797**	**n = 1218**		**n = 421**	
Mean age	68.63	68.5		0.75	68.4	0.67
Mean PSA	8.47	8.18		0.28	8.12	0.252
Gleason Score (mean)	6.9	6.38		<0.001	6.83	0.99
Race				<0.001		0.042
White	278 (11.7%)	650 (81.6)	928		313 (74.3%)	
Black	79 (18.8%)	96 (12%)	175		67 (15.9%)	
Hispanic	44 (10.4%)	34 (4.3%)	78		27 (6.4%)	
Other	20 (4.8%)	17 (2.1%)	37		14 (3.3%)	
Year of diagnosis (mean)	2003	1997		<0.001	2001	<0.001
Dose of radiation				<0.001		
Low ≤ 70	28 (6.6%)	390 (49.8%)	418		71 (16.9%)	<0.001
High > 70	393 (93.3%)	407 (51.1%)	800		350 (8.3%)	
Clinical T-stage				0.27		0.47
T1	205 (48.7%)	336 (42.2%)	571		234 (55.6%)	
T2a	94 (22.3%)	164 (20.1%)	258		95 (22.6%)	
T2b,c	122 (30%)	267 (33.5%)	389		92 (21.8%)	
Length of concurrent hormones*						
Short (≤6 months)	271 (64.4%)		271			
Long (>6 months)	150 (35.6%)		150			
Timing of hormones						
Neoadjuvant	371 (88.1%)		371			
Adjuvant	50 (11.9%)		50			
Salvage	8 (1.9%)	114 (14.3%)	122	<0.001	40 (9.5%)	<0.001
Radiation type<						<0.001
3-D conformal	73 (17.3%)	546 (68.5%)	619	<0.001	171 (40.6%)	
IMRT	348 (82.7%)	251 (31.5%)	599		250 (59.4%)	

*(of patients who received adjuvant hormones).

Percentages may not sum to 100% due to rounding.

As expected, there were significant differences in baseline characteristics between the unmatched groups that received ADT and those that did not receive ADT. No difference was noted in age, PSA, or T-stage, but patients who did not receive ADT had lower mean Gleason scores, earlier years of diagnosis, lower doses of radiation, more biochemical and distant failures. About half (51%) were treated with 3D conformal radiation, but the type of radiation received was found to be highly correlated with radiation dose, as 598 of the 599 patients who were treated with IMRT were also given high dose radiation. As such, radiation technique was not evaluated as a variable for the remainder of the analyses.

When comparing the cohort of men who received ADT to the matched controls (Table 1), no difference was noted in age, PSA, Gleason score, or T-stage, but patients who did not receive ADT had earlier years of diagnosis (*P* <0.001), lower doses of radiation, more biochemical and distant failures, were more likely to be white, and were more likely to be placed on salvage hormone therapy than patients who received ADT (*P* <0.001). Matching appeared to resolve the difference in Gleason score that was seen in the unmatched dataset.

On comparing the five and ten year rates of PSA failure by ADT status and radiation dose (Table 2), there is a significant difference in the groups (*P* <0.001 for both five and ten year failures).

**Table 2:** **Univariable analysis of dose by ADT status**

	**ADT status**		
**Radiation dose**	**ADT**	**No ADT**	p-values*
High (>70)			
Five year failure	4.10%	8.60%	
Ten year failure	5.60%	11.10%	
Low ≤ 70)			
Five year failure	3.60%	16.90%	
Ten year failure	7.10%	32.60%	

*p < 0.001 for both five and ten year failures.

On univariable analyses for biochemical failure, higher PSA (HR, 1.075; 95% CI, 1.045- 1.106; *P* = <0.001), lower Gleason Score (HR, 0.863; 95% CI, 0.761-0.978; *P* = <0.021), earlier year of diagnosis (HR, 0.902; 95% CI, 0.875-0.930; *P* <0.001), higher T-stage (HR, 1.215; 95% CI, 1.043-1.416; *P* =0.012), lower doses of radiation (HR, 0.431; 95% CI, 0.320-0.581; *P* <0.001), and no ADT (HR, 0.422; 95% CI, 0.274-0.651; *P* <0.001) were associated with an increased risk of biochemical failure (Table 3). No difference in biochemical failure was seen among different racial groups, neoadjuvant hormone use (HR, 1.514; 95% CI, 0.444-5.160; p = 0.507) or with the use of greater than 6 months of ADT compared with less than 6 months (HR, 0.571; 95% CI, 0.23-1.416; *P* =0.226).

**Table 3:** **Univariable analysis of association with biochemical failure and distant metastasis**

	**Biochemical failure**			**Distant metastasis**		
**Variable**	**Hazard**	**95% CI**	**p-value**	**Hazard**	**95% CI**	**p-value**
	**Ratio**			**Ratio**		
Age	1.000	0.980-1.02	0.983	1.016	0.968-1.067	0.522
Pre-treatment PSA	1.075	1.045-1.106	<0.001	1.043	0.975-1.115	0.22
Gleason Score	0.863	0.761-0.978	0.021	1.100	0.796-1.519	0.564
Year of Diagnosis	0.902	0.875-0.930	<0.001	0.926	0.858-0.999	0.046
T-stage	1.215	1.043-1.416	0.012	1.668	1.125-2.474	0.011
Radiation Dose (high vs low)	0.431	0.320-0.581	<0.001	0.593	0.287-1.226	0.158
ADT use (yes vs no)	0.422	0.274-0.651	<0.001	0.119	0.016-0.882	0.037
Length of Hormone use	0.981	0.938-1.026	0.403	1.000	0.914-1.094	0.997
Race	0.795	0.611-1.034	0.087	0.678	0.318-1.444	0.263
ADT Timing (Neoadjuvant vs adjuvant)	1.514	0.444-5.160	0.507	1.047	0.408-2.688	0.924

Univariable analysis for distant metastasis (Table 3) showed that earlier year of diagnosis (HR, 0.926; 95% CI, 0.858-0.999; *P* =0.046), higher T-stage (HR, 1.668; 95% CI, 1.125-2.474; *P* =0.011), and the lack of ADT (HR, 0.119; 95% CI, 0.016-0.882; *P* =0.037) were associated with an increased risk of distant metastasis. No difference in distant failure (Table 3) was seen with age (HR, 1.016, 95% CI, 0.968-1.067; *P* =0.522), Pre-treatment PSA (HR, 1.043; 95% CI, 0.975-1.115; *P* =0.22), Gleason score (HR, 1.1; 95% CI, 0.796-1.519; *P* =0.564), race (HR, 0.678; 95% CI, 0.318-1.444; *P* =0.263) or with the use of greater than 6 months of ADT compared with less than 6 months (HR, 1.00; 95% CI, 0.914-1.094; *P* =0.997). The length of ADT and race were not statistically significant. A Cox model for biochemical failure was constructed and shown as the “Initial Cox” column in Table 3. Variables that were significant in the univariable analyses were selected for inclusion in this model. The adjusted model showed that higher pre-treatment PSA (HR, 1.079; 95% CI, 1.042-1.111; *P* <0.001), higher Gleason Score (HR, 1.118; 95% CI, 1.012-1.393; *P* =0.026), earlier year of diagnosis (HR, 0.923; 95% CI, 0.875-1.373; *P* = 0.004), higher T-stage (HR, 1.668; 95% CI, 1.125-2.474; *P* = 0.011), and the lack of ADT (HR, 0.599; 95% CI, 0.364- 0.978; *P* =0.04) were associated with an increased risk of biochemical failure while controlling for these and the additional variables included in the model (age, T-stage, and radiation dose). The use of ADT was associated with a lower risk of biochemical failure as compared to that of no ADT (HR, 0.599; 95% CI, 0.367-0.978; *P* <0.04). An interaction term of adjuvant hormone use and radiation dose was not significant when added to the model (HR, 3.883; 95% CI, 0.873- 17.26; *P* =0.075), meaning that radiation dose is not an effect modifier. A subsequent multivariate Cox proportional hazard model was performed after matching (Table 4, column titled “Matched Cox Analysis”).

**Table 4:** **Cox models for biochemical failure and distant metastasis**

	**Biochemical failure**						**Distant metastasis**		
	**Initial cox**			**Matched cox analysis**			**Initial cox**		
**Variable**	**Hazard**	**95% CI**	**p-value**	**Hazard**	**95% CI**	**p-value**	**Hazard**	**95% CI**	**p-value**
	**Ratio**			**Ratio**					
Age	1.003	0.983-1.024	0.76	0.983	0.955-1.011	0.235	1.022	0.972-1.074	0.393
Pre-treatment PSA	1.079	1.042-1.111	<0.001	1.055	0.997-1.116	0.062	1.077	1.004-1.155	0.039
Gleason Score	1.118	1.021-1.393	0.026	1.996	1.045-3.812	0.036	1.480	1.020-2.149	0.039
Year of diagnosis	0.923	0.875-9.74	0.004	0.992	0.907-1.085	0.867	0.969	0.856-1.098	0.623
T-stage	1.164	0.987-1.373	0.072	1.371	1.058-1.774	0.017	1.820	1.201-2.759	0.005
Radiation dose (high vs low)	0.864	0.572-1.306	0.489	0.928	0.490-1.757	0.818	1.039	0.413-2.609	0.936
Adjuvant Hormone use (yes vs no)	0.599	0.364-0.978	0.04	0.487	0.288-0.822	0.007	0.114	0.014-0.905	0.04
Interaction term ADT x XRT	3.883	0.873-17.26	0.075						

A Cox model for distant metastasis was constructed and shown as the “Initial Cox” column in Table 4. Variables that were significant in the univariable analyses were selected for inclusion in this model. The initial Cox model showed that higher pre-treatment PSA (HR, 1.077; 95% CI, 1.004-1.155; *P* =0.039), higher Gleason Score (HR, 1.480; 95% CI, 1.020-2.149; *P* =0.039), higher T-stage (HR, 1.820; 95% CI, 1.201-2.759; *P* =0.005), and the lack of ADT (HR, 0.114; 95% CI, 0.014-0.905; *P* =0.04) were associated with an increased risk of distant metastasis while controlling for these and the additional variables included in the model, such as Age, year of diagnosis, and radiation dose. The main outcome from the initial Cox, shows that the use of ADT was associated with a lower risk of distant metastasis than no ADT. The effect of the main variable, ADT use in both the initial and matched Cox models were that the use of ADT was associated with decreased risk of biochemical failure (HR, 0.599; 95% CI, 0.364-0.978; *P* = 0.04 for the initial Cox, (HR, 0.487; 95% CI 0.228-0.822, *P* = 0.007 for the stratified Cox).

In addition, two other techniques with the propensity score (stratification by propensity score and using the propensity score as a covariate) were used as a sensitivity analysis to validate these results [16]. The quintiles of the propensity scores were used to stratify patients into five homogeneous groups with respect to their likelihood of being given ADT (the propensity score). A stratified Cox regression analysis based on the propensity score was then conducted with separate estimates for all of the variables in each of the five strata. A weighted average of the stratum-specific estimates was then calculated (Table 5, “stratified Cox column”).

**Table 5:** **Cox models for biochemical failure: initial, stratified, PS as covariate**

	**Initial cox**			**Stratified by PS quintiles**			**Cox with PS as covariate**		
**Variable**	**Hazard**	**95% CI**	**p-value**	**Hazard**	**95% CI**	**p-value**	**Hazard**	**95% CI**	**p-value**
	**Ratio**			**Ratio**					
Age	1.003	0.983-1.024	0.76	1.005	0.984-1.026	0.666	1.004	0.984-1.025	0.684
Pre-treatment PSA	1.079	1.042-1.111	<0.001	1.065	1.026-1.105	0.001	1.056	1.020-1.093	0.002
Gleason Score	1.118	1.021-1.393	0.026	1.187	0.997-1.414	0.054	1.13	0.968-1.319	0.122
Year of diagnosis	0.923	0.875-9.74	0.004	0.888	0.821-0.961	0.003	0.875	0.816-0.941	<0.001
T-stage	1.164	0.987-1.373	0.072	1.09	0.899-1.323	0.379	1.053	0.873-1.272	0.588
Radiation dose (high vs low)	0.864	0.572-1.306	0.489	0.835	0.541-1.290	0.417	0.845	0.552-1.272	0.44
Adjuvant hormone use (yes vs no)	0.599	0.364-0.978	0.04	0.483	0.287-0.813	0.006	0.492	0.292-0.829	0.008
Propensity Score							4.679	1.172-18.682	0.029

Stratifying the patients into propensity score quintiles resulted in an improved balancing of patient characteristics between the ADT/no ADT groups compared to the initial differences prior to stratification seen in Table 1. After stratification, the only significant differences remaining within the ADT/no ADT groups after dividing into propensity score quintiles were the year of diagnosis in Quintile 2 and 3 (p = 0.013 and <0.001), the T-stage in quintiles 2 (P = 0.032), 3 (p = 0.01) and 5 (p < 0.001), the radiation dose in quintile 3 (p = 0.019), and the number of failures in quintile 2 (p = 0.034) and 3 (p = 0.016). Finally, a Cox multivariable analysis for the outcome of biochemical failure was conducted within each propensity score quintile and the weighted average was calculated and presented in Table 3. Due to the small number of distant metastasis, a stratified analysis was not done for that outcome. Finally, a Cox model was run which included the propensity score as a covariate (Table 6, column “Cox with PS as Covariate”).

**Table 6:** **Cox models for distant metastasis: initial, PS as covariate**

	**Initial cox**			**Cox with PS as covariate**		
**Variable**	**Hazard**	**95% CI**	**p-value**	**Hazard**	**95% CI**	**p-value**
	**Ratio**			**Ratio**		
Age	1.022	0.972-1.074	0.393	1.022	0.972-1.074	0.395
Pre-treatment PSA	1.077	1.004-1.155	0.039	1.078	0.986-1.179	0.098
Gleason Score	1.48	1.020-2.149	0.039	1.488	0.994-2.225	0.05
Year of diagnosis	0.969	0.856-1.098	0.623	0.973	0.815-1.163	0.765
T-stage	1.82	1.201-2.759	0.005	1.833	1.151-2.917	0.011
Radiation dose (high vs low)	1.039	0.413-2.609	0.936	1.038	0.414-2.604	0.936
Adjuvant hormone use (yes vs no)	0.114	0.014-0.905	0.04	0.116	0.014-0.985	0.048
Propensity Score				0.889	0.023-34.367	0.95

The effect of the main variable, ADT use in all of the models (in addition to the matched analysis shown in Table 3 of the paper) was that the use of ADT decreases the risk of biochemical failure (HR, 0.599; 95% CI, 0.364-0.978; *P* = 0.04 for the initial Cox, HR, 0.483; 95% CI, 0.287-0.813; *P* = 0.006 for the stratified Cox, and HR, 0.492; 95% CI, 0.292-0.829; *P* = 0.008 for the model with propensity scores as a covariate). This study showed that all three of the adjusted models showed a similar result as the unadjusted Cox model; there is a significant benefit to the use of adjuvant hormone therapy in reducing biochemical failures.

A Cox model for distant failure was constructed and shown as the “Initial Cox” column in Table 2. Variables that were significant in the univariable analyses were selected for inclusion in this model. The initial Cox model showed that higher pre-treatment PSA (HR, 1.077; 95% CI, 1.004-1.155; *P* = 0.039), higher Gleason Score (HR, 1.48; 95% CI, 1.020-2.149; *P* = 0.039), higher T-stage (HR, 1.82; 95% CI, 1.201-2.759; *P* = 0.005), and the lack of ADT (HR, 0.114; 95% CI, 0.014-0.905; *P* = 0.04) were associated with an increased risk of biochemical failure while controlling for these and the additional variables included in the model, such as Age, year of diagnosis, and radiation dose. The main outcome from the initial Cox, shows that the use of ADT was associated with a lower risk of distant failure than no ADT.

Due to the small number of distant failures, a matched and stratified analysis was not done for this outcome, but a Cox model was run which included the propensity score as a covariate (Table 5, column “Cox with PS as Covariate”). The effect of the main variable, ADT use in all Cox models was that the use of ADT decreases the risk of distant failure (HR, 0.114; 95% CI, 0.014-0.905; *P* = 0.04 for the initial Cox, and HR, 0.16; 95% CI, 0.014-0.985; *P* = 0.048 for the model with PS as a covariate). Both of the models showed a similar result; there is a significant benefit to the use of adjuvant hormone therapy in reducing distant failures.

## Conclusions

Our study showed that the addition of ADT to external beam radiation was associated with a significantly increased PSA-free survival in intermediate risk patients. This benefit held when controlling for other known prognostic factors (age, PSA, Gleason score, year of diagnosis, T-stage, and the dose of radiation) and after including propensity scores. However, our study did not show a benefit to giving longer than 6 months of ADT.

Several trials have been conducted to evaluate the benefits of combining radiation and ADT in intermediate risk patients. In the RTOG 86-10 study, Pilepich et al. evaluated 471 patients from 1987 to 1991 with clinical stage T2-T4 with or without lymph node metastasis and randomized them to radiation therapy (65-70 Gy) alone versus radiation therapy with hormone therapy (goserelin and flutamide) for two months before and during radiation therapy [2,17]. At 10 years of follow up, the combined group showed an overall survival of 43% compared with the radiation therapy only arm of 34%, which was not statistically significant. However, statistically significant improvements in disease-specific mortality (23% vs 36%, *P* = 0.01), distant metastasis (35% vs 47%, *P* = 0.006), and biochemical failure (65% vs 80%, *P* <0.001) were seen on the hormone therapy arm. D’Amico et al. evaluated intermediate and high risk patients who received 70 Gy +/- 6 months of ADT and found that ADT resulted in an improvement in overall survival (74% vs 61%, *P* = 0.01) [15].

Once the benefit of adding hormone therapy to radiation therapy was established, researchers began to investigate the optimum duration of hormone therapy by shortening the regimens and comparing to more protracted regimens, and as in our study, found no benefit to giving longer courses of ADT. The Irish Clinical Oncology Research Group 97-01 study was conducted from 1997 to 2001 [18]. This study randomized 261 patients with localized, node negative, intermediate to high risk, PSA > 20 disease to 70 Gy of radiation with either a short (4 month) or long (8 month) course of neoadjuvant hormone therapy (LHRH with flutamide). At 102 months of follow-up, there was no statistically significant difference between the two groups in terms of overall survival, biochemical-free survival, or cancer-specific survival. The Canadian Multicenter study was conducted from 1995 to 2001 [19]. A total of 378 men with clinically localized cT1-T4 (43% intermediate risk) were randomized to receive either 3 or 8 months of hormone therapy (flutamide and goserelin) prior to definitive radiation to 66 Gy. Overall, no difference was seen in biochemical failure or patterns of failure between both arms in intermediate risk patients. D’Amico et al. analyzed a total of 311 men with a median age of 70, who had been enrolled on 3 prospective randomized trials from 1987-2000 who received either 6 months or 3 years of hormone therapy in addition to definitive radiation therapy [15]. Radiation doses were between 66 and 70 Gy and hormone therapy was given either as combined androgen blockade and or from single-agent therapy only. They found that after adjusting for known prognostic factors, the use of 3 years of hormone therapy did not improve survival compared to 6 months of hormone therapy.

No trials have yet been completed which incorporate higher doses of radiation and the current risk classification as per NCCN guidelines. The current trial, RTOG 99-10, does use modern risk stratification schemes, comparing 8 weeks versus 28 weeks of neoadjuvant androgen suppression followed by a 70 Gy dose of radiation with 8 weeks of concurrent ADT and closed for accrual in May of 2004. The preliminary results, presented in abstract form, suggest no improvement of the endpoints of biochemical, loco-regional, or distant relapse or death with extending the neoadjuvant androgen suppression [20]. A retrospective study focusing on patients treated in the modern era (1993-2008) was conducted at our own institution, and found that in unfavorable patients (defined as Gleason 4 + 3 or T2c), the addition of ADT provided an improvement in freedom from failure (74% with no ADT vs 94% with ADT at 5 years, *P* = 0.0049) [21]. The GETUG 14 randomized trial evaluated high dose radiotherapy of 80 Gy alone or in combination with 4 months of ADT, and was closed prematurely due to slow accrual, but intermediary analysis did not reach a statistical significance [22,23].

Based on our findings, it appears that it is reasonable to consider 4-6 months of ADT for intermediate risk patients. Since intermediate risk prostate cancer is a heterogeneous group, it is also reasonable to consider the disease burden and comorbidities to assist in making the clinical decisions regarding ADT in view of the higher doses currently applied.

Strengths of our study are a large cohort of patients who were consistently treated during a given period, long follow up, and pathology reviewed at a single institution. An additional strength is that the method of propensity scores was used to reduce bias in this retrospective study design. When a matched analysis was conducted as a sensitivity test to reduce bias using the propensity score, the results showed that a benefit still existed to the addition of ADT while controlling for other prognostic factors. The major limitations in our study are apparent in the non-randomized nature of patients receiving and not receiving ADT. This bias is seen in the discrepancy in baseline PSA values, Gleason scores, and t-stage, and efforts were made to minimize the bias by use of the propensity score. However, some of the imbalances seen favored patients that did not receive ADT, i.e., lower Gleason score and lower PSA. Despite this, patients who did not receive ADT had increased biochemical failures. As such, it is likely that ADT does have a significant biologic effect in reduction of biochemical failures. In our study, the duration and type of ADT usage was typical for the time period but not standardized. Additionally, this retrospective study analysis included only patients treated in a tertiary cancer center, and comorbidities and obesity were not at the time included in the data elements, all of which may limit the generalizability.

An overall improvement in PSA recurrence-free survival and distant metastasis-free survival was associated with the use of ADT while controlling for age, T-stage, PSA, year of diagnosis, and Gleason scores in intermediate risk prostate cancer. There was no apparent reduction in biochemical failure by giving longer than 6 months duration of ADT. Randomized trials are in progress to further define the benefit of androgen deprivation therapy with high dose external beam radiation in intermediate risk disease.
